# Understanding Changes in Serum Creatinine During Work in Heat

**DOI:** 10.1016/j.ekir.2025.04.047

**Published:** 2025-04-26

**Authors:** Erik Hansson, Rebekah A.I. Lucas, Jason R. Glaser, Ilana Weiss, Ulf Ekström, Magnus Abrahamson, Catharina Wesseling, David H. Wegman, Kristina Jakobsson

**Affiliations:** 1 Occupational and Environmental Medicine, School of Public Health and Community Medicine, University of Gothenburg, Gothenburg, Sweden; 2 La Isla Network, Washington DC, USA; 3 School of Sport, Exercise and Rehabilitation Sciences, University of Birmingham, Birmingham, UK; 4 Division of Clinical Chemistry and Pharmacology, Department of Laboratory Medicine, Lund University, Sweden; 5 Department of Public Health, University of Massachusetts Lowell, Lowell, Massachusetts, USA

**Keywords:** creatinine, cystatin C, heat

See Commentary on Page 2527

## Introduction

Repeated kidney injury from strenuous work in heat is increasingly linked to chronic kidney disease of nontraditional origin (CKDnt).^1^ Several studies have reported pronounced increases in serum creatinine (sCr) levels after strenuous work performed under hot environmental conditions in populations at risk of CKDnt.^2^, ^3^, ^4^, ^5^, ^6^, ^7^^,^S1^,^S2 However, it remains unclear if such changes truly represent decreased glomerular filtration rate (GFR), or instead reflect reduced plasma volume, or increased muscle metabolism resulting in increased creatinine production.^4^^,^^5^ We have previously described a high correlation between changes in estimated GFR based on sCr and serum cystatin C (sCysC, a GFR marker independent of muscle metabolism) during a harvest season.^S3^ However, among Spanish berry pickers, likely exposed to less heat stress than sugarcane workers at risk of CKDnt, those with cross-shift sCr increase > 0.3 mg/dl had change in sCysC (ΔsCysC) similar to those with stable sCr.^5^

The aims of the study were as follows: (i) assess whether cross-shift changes in sCr (ΔsCr), as a routinely available GFR marker, is associated with ΔsCysC; (ii) assess whether these markers increase when considering plasma volume changes (ΔPV) during physically heavy work in heat; and (iii) describe the relationship between ΔsCr, ΔsCysC, and changes in serum creatine kinase (ΔsCK), as a marker of muscle injury and work.

We explored these questions in datasets from two previously reported cross-shift studies conducted among Nicaraguan^6^ and Salvadoran^7^ sugarcane workers sampled before and after performing physically strenuous work (average heart rate: 59%S4 of maximum) in hot environments (wet bulb globe temperatures: ∼30°CS5). Nicaraguan workers had a well-developed rest-shade-hydration (RSH) intervention,^6^^,^S5 whereas the Salvadoran workers received an intervention during the second half of the study period.^7^

**Figure 1:**
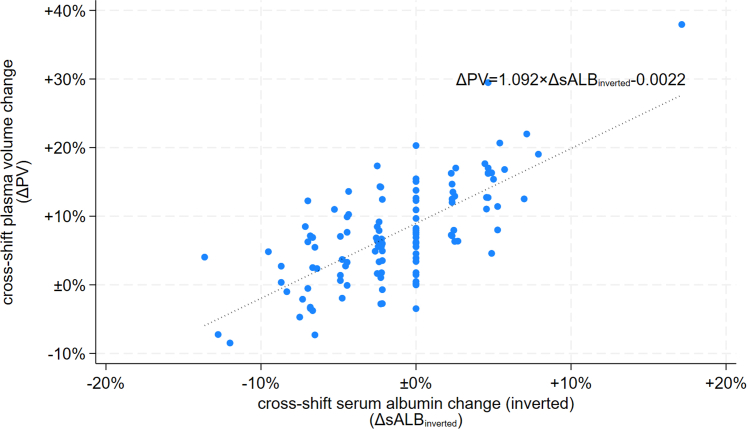
Association between cross-shift changes in plasma volume (estimated using the Dill and Costill^8^ equation) and serum albumin concentrations in the Salvadoran cohort.

ΔPV during exercise is typically evaluated using the Dill and Costill 1974 equation,^8^ based on blood hemoglobin and hematocrit before and after exercise, which was lacking for the Nicaraguan group. Albumin concentration was analyzed in both cohorts in stored frozen serum samples. The rationale for using albumin concentration as a marker of ΔPV was that it is a primarily intravascularly located protein with a long half-life (3 weeks) and a relatively stable expression outside of severe inflammatory conditions and malnutrition. Cross-shift albumin concentration changes (ΔsALB) are therefore likely due to changes in plasma volume.S6 Previous research has found that changes in ΔsALB strongly correlate with ΔPV estimated using the Dill and Costill equation,^8^^,^^9^^,^S7 and this was the case in our Salvadoran dataset with hematological data and serum albumin (Figure 1). We therefore used ΔsALB to estimate ΔPV and to correct cross-shift serum concentrations for ΔPV (Supplementary Methods).

## Results

**Figure 2:**
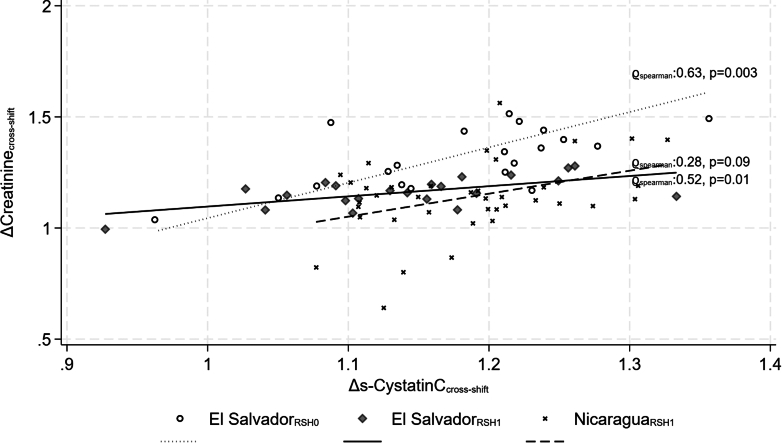
Associations between cross-shift changes in serum creatinine and cystatin C. Cross-shift concentration changes have been adjusted for plasma volume concentration changes (Figure 1). One observation at ΔsCr = 2.12 and ΔsCysC = 1.50 is not shown for graphical purposes. ΔsCR, change in serum creatinine; ΔsCysC, change in serum cystatin C; RSH0, no rest-shade-hydration intervention; RSH1, rest-shade-hydration intervention implemented.

ΔsALB strongly correlated with ΔPV within the Salvadoran dataset^8^ (Figure 1). Positive ΔPV indicates that workers generally had an increasing plasma volume during work shifts. Despite this, both sCr and sCysC increased during the work shift in most workers (Figure 2). ΔsCr and ΔsCysC correlated with each other (Figure 2), at ϱ = 0.63 (*P* = 0.003) and 0.52 (*P* = 0.01) in the Salvadoran workers before and shortly after implementing an early stage RSH intervention, and ϱ = 0.28 (*P* = 0.09) in the Nicaraguan workers with a well-implemented RSH intervention (Figure 2).

## Discussion

Increasing sCr during heavy work in heat without RSH was not explained by reduced plasma volume and was mirrored by increasing sCysC. Further, correlations between ΔsCysC and ΔsCK were similar to correlations between ΔsCr and ΔsCK (Supplementary Figures S1 and S2). This indicates that increasing sCr during heavy work in heat among populations at risk of CKDnt reflects reduced GFR, rather than muscle work/breakdown *per se*. Notably, the majority of workers in the current study appeared to drink sufficient quantities throughout their work shift so that their plasma volume expanded, with cross-shift ΔPV increase and decrease in albumin concentration correlating with corresponding body weight and serum sodium concentration changes (Supplementary Figure S3).

Marked increase in sCr (> 0.3 mg/dl) across the work shift is sometimes reported as “acute kidney injury.”^4^^,^^5^^,^S8 Whether such increase truly reflects episodes of structural kidney injury remains unclear, with sCr often normalizing the subsequent morning.^6^ We propose to consider “kidney strain” as an alternative expression for this response, emphasizing the temporary and largely reversible nature of this heat stress response. Moreover, it indicates the need to mitigate the stressor in the workforce, rather than the patient-centered clinical management of a dichotomous “injury” event.

Associations between ΔsCr, ΔsCysC, and ΔsCK differed between the settings, potentially because of the Nicaraguan workers having a well-implemented RSH intervention whereas these were absent or just beginning to be implemented in El Salvador. Preventing heat stress and lowering kidney strain would be expected to lead to less pronounced associations between these biomarkers, as was observed (Supplementary Figures S2 and S3). Productivity levels remained or even increased with RSH intervention in Nicaragua,S9 and ΔsCK remained elevated, indicating muscle work was still strenuous. However, ΔsCK was not positively linked to ΔsCr or ΔsCysC in Nicaragua, which tentatively may be because of the effects of muscle heat production or muscle breakdown becoming smaller when an advanced RSH program was well-implemented.

Cystatin C does not increase together with acute inflammation markers after elective surgeryS10; however, it has been associated with inflammation markers in epidemiological studies.S11 It thereby seems unlikely that the rapid increases in cystatin C observed are due to acute inflammation. However, considering the high levels of inflammation in these workers and the proinflammatory potential of heat stress,^2^ we will continue to study the relationship between inflammation and kidney injury during heat stress.

Sugarcane workers are at a high risk of CKDnt, and the repeated kidney strain described here may be a cause. The design of this study does not allow for linking daily fluctuations in GFR to long-term kidney outcomes. One previous study has reported that pronounced daily fluctuations in sCr was related to worse kidney outcomes during the harvest season at an individual level.^4^ Other markers than the ones based on renal glomerular function, such as urinary proteins specific to tubular injuryS12 or urinary tract inflammation,S13 may be useful for detecting important kidney strain/injury during heat stress.

The substitute correction method based on ΔsALB for estimating ΔPV may have introduced some, likely nondifferential error. Considering similar results when adjusting for ΔPV based on hematological data rather than albumin in the Salvadoran dataset (Supplementary Figure S4), the magnitude is probably small. Being able to estimate ΔPV in stored samples and not rely on fresh-sample hematological analysis is useful for field studies.

Our findings again^6^ stress the need to consistently sample participants before work shifts in prevalence studies of kidney function, for example, DEGREE-protocol studiesS14 and longitudinal studies of workers. This is especially important when the work involves substantial heat stress because postshift sCr are unlikely to reflect steady-state GFR.

In conclusion, ΔsCr during physically demanding work in heat reflects reduced glomerular filtration rather than decreased plasma volume or increased muscle metabolism. It merits further consideration, especially as a group-level marker of potentially harmful working conditions with repetitive kidney strain/injury in the context of heat-related nephropathy.

## Disclosure

All the authors declared no competing interests.

## Supplementary Material

Supplementary File (PDF) **Supplementary Methods. Supplementary References. Figure S1.** Associations between pre-to-post shift changes in serum creatinine and serum creatine kinase. **Figure S2.** Associations between pre-to-post shift changes in serum cystatin C and serum creatine kinase. **Figure S3.** Association between pre-to-post shift changes in markers of fluid balance in Salvadoran workers. **Figure S4.** Associations between cross-shift changes in serum creatinine and cystatin C in the Salvadoran cohort, when adjusting for plasma volume changes using the Dill and Costill equation.^8^

## Footnotes

Supplementary File (PDF)

**Supplementary Methods.**

**Supplementary References.**
**Figure S1.** Associations between pre-to-post shift changes in serum creatinine and serum creatine kinase. **Figure S2.** Associations between pre-to-post shift changes in serum cystatin C and serum creatine kinase. **Figure S3.** Association between pre-to-post shift changes in markers of fluid balance in Salvadoran workers. **Figure S4.** Associations between cross-shift changes in serum creatinine and cystatin C in the Salvadoran cohort, when adjusting for plasma volume changes using the Dill and Costill equation.^8^

## References

[1] Wesseling C., Glaser J., Rodríguez-Guzmán J. (2020). Chronic kidney disease of non-traditional origin in Mesoamerica: a disease primarily driven by occupational heat stress. Pan Am J Public Health.

[2] Hansson E., Glaser J., Jakobsson K. (2020). Pathophysiological mechanisms by which heat stress potentially induces kidney inflammation and chronic kidney disease in sugarcane workers. Nutrients.

[3] Butler-Dawson J., Krisher L., Yoder H. (2019). Evaluation of heat stress and cumulative incidence of acute kidney injury in sugarcane workers in Guatemala. Int Arch Occup Environ Health.

[4] Dally M., Butler-Dawson J., Johnson R.J. (2020). Creatinine fluctuations forecast cross-harvest kidney function decline among sugarcane workers in Guatemala. Kidney Int Rep.

[5] Koch S., Buekers J., Espinosa A. (2025). Association between objectively assessed physical activity and kidney function among female agricultural workers in hot environments in Spain. Environ Res.

[6] Lucas R.A.I., Hansson E., Skinner B.D. (2025). The work-recovery cycle of kidney strain and inflammation in sugarcane workers following repeat heat exposure at work and at home. Eur J Appl Physiol.

[7] Wegman D.H., Apelqvist J., Bottai M. (2018). Intervention to diminish dehydration and kidney damage among sugarcane workers. Scand J Work Environ Health.

[8] Dill D.B., Costill D.L. (1974). Calculation of percentage changes in volumes of blood, plasma, and red cells in dehydration. J Appl Physiol.

[9] Miller G.D., Teramoto M., Smeal S.J., Cushman D., Eichner D. (2019). Assessing serum albumin concentration following exercise-induced fluid shifts in the context of the athlete biological passport. Drug Test Anal.

