# Supplementary material for: Understanding Changes in Serum Creatinine During Work in Heat
Source: Kidney Int Rep. 2025 Apr 26;10(8):2860–3. doi: 10.1016/j.ekir.2025.04.047 (PMC12348246; doi:10.1016/j.ekir.2025.04.047)
Supplement: Supplementary File (PDF) — Supplementary Methods. Supplementary References. Figure S1. Associations between pre-to-post shift changes in serum creatinine and serum creatine kinase. Figure S2. Associations between pre-to-post shift changes in serum cystatin C and serum creatine kinase. Figure S3. Association between pre-to-post shift changes in markers of fluid balance in Salvadoran workers. Figure S4. Associations between cross-shift changes in serum creatinine and cystatin C in the Salvadoran cohort, when adjusting for plasma volume changes using the Dill and Costill equation.8 [file mmc1.pdf]

Supplement Methods

Study settings and participants

El Salvador

Male sugarcane cutters were sampled before and after work shifts, a couple of months into the sugarcane harvest season 2014/2015. Sampling and laboratory procedures have been reported previously [7] but briefly, blood samples were placed on ice immediately and serum was frozen within hours of collection and transported at -80°C to Lund University Hospital, Sweden, for serum creatinine and albumin analysis using a Roche Cobas 701 (Roche Diagnostics, Basel, Switzerland). Blood analyses for complete blood counts including hemoglobin and haematocrit were performed on fresh samples at CECIAM Escalón, San Salvador. Serum cystatin C was also analyzed on Cobas instruments at Lund University Hospital in a subsample of workers. The inclusion criteria for that group were that participating workers were male, had two complete cross-shift (pre- and post-) measurements and were ≤46 years old.

Nicaragua

A convenience sample of 20 male sugarcane cutters had serum samples collected before and after workshifts on a Saturday and the subsequent Monday a couple of months into the 2020 sugarcane season [6]. This convenience sample was composed of workers participating in the third year of the Adelante/PREP intervention study, when the rest-shade-hydration intervention had been enhanced [S5]. Laboratory procedures have been reported previously [6] but briefly, blood samples were placed on ice immediately and serum was frozen within hours of collection and transported at -80°C to Lund University, Sweden, for serum creatinine, cystatin C and albumin analysis using a Cobas 701 instrument. Complete blood count analyses were not performed. We excluded two cross-shift observations with physiologically implausible cross-shift changes in serum creatinine or cystatin C, which were > 4 SD different from the rest of the observations and interpreted as laboratory errors [6].

Statistical analysis

All statistical analyses were performed using Stata 18. For both settings, non-parametric correlation coefficient between plasma volume-corrected changes in sCr, sCysC and sCK across the work shift were estimated using the *spearman* command. Corrections for cross-shift changes in plasma volume were made as described below.

Plasma volume changes across the work shift (ΔPV) in the Salvadoran dataset, which had the necessary hematological parameters, was calculated using the Dill and Costill 1974 equation [8]. A linear regression model was used to predict ΔPV based on ΔsALB. Visual inspection of the normality of residuals and the homoscedasticity of residuals versus predicted values were used to assess fulfillment of model assumptions. The coefficients from this model were applied to both cohorts, in order to estimate comparable plasma volume changes for use in the main analyses.

Supplement Figures

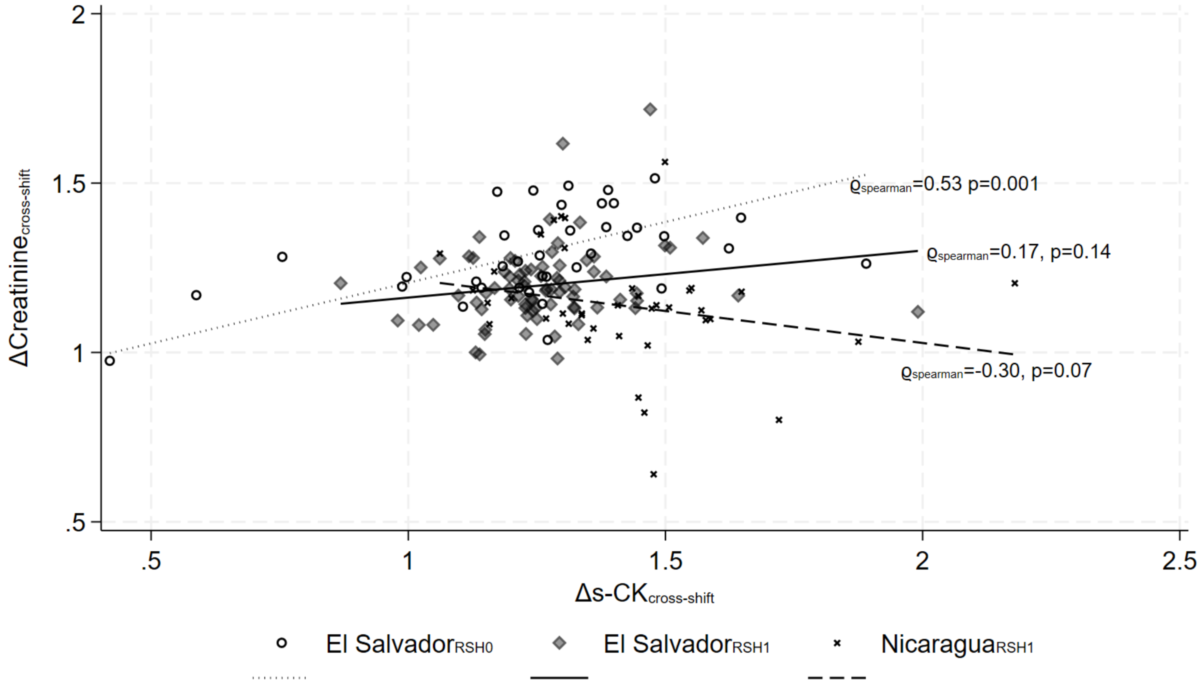

Supplement Figure 1. Associations between pre-to-post shift changes in serum creatinine and serum creatine kinase.

RSH0 = No rest-shade-hydration intervention. RSH1 = rest-shade-hydration intervention implemented. Cross-shift concentration changes have been adjusted for plasma volume concentration changes (Figure 1). One observation at ΔsCr=2.12 and ΔsCK=3.01 is not shown for graphical purposes.

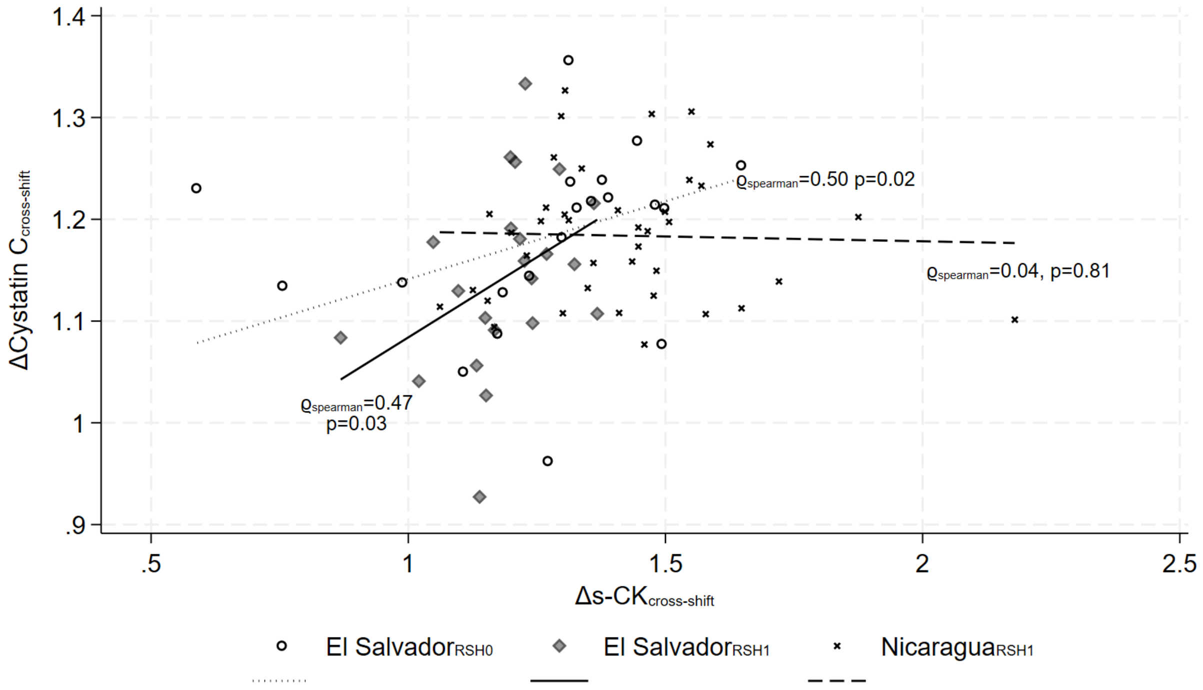

Supplement Figure 2. Associations between pre-to-post shift changes in serum cystatin C and serum creatine kinase.

RSH0 = No rest-shade-hydration intervention. RSH1 = rest-shade-hydration intervention implemented. Cross-shift concentration changes have been adjusted for plasma volume concentration changes (Figure 1). One observation at ΔsCysC=1.50 and ΔsCK=3.01 is not shown for graphical purposes.

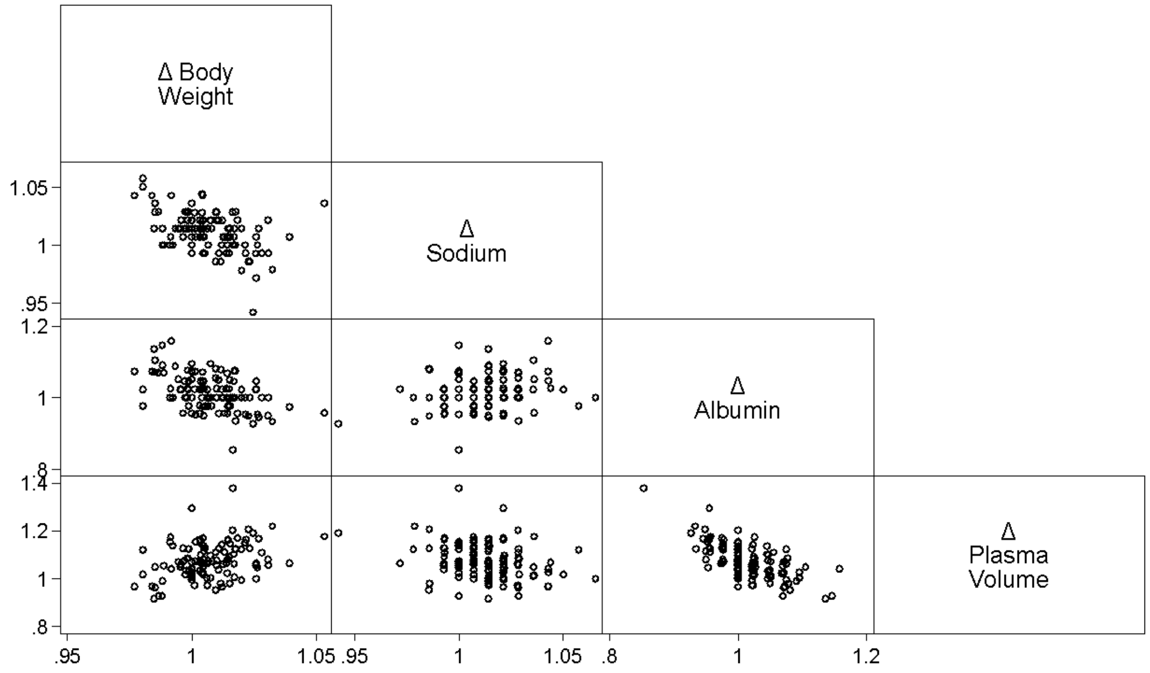

Supplement Figure 3. Association between pre-to-post shift changes in markers of fluid balance in Salvadoran workers.

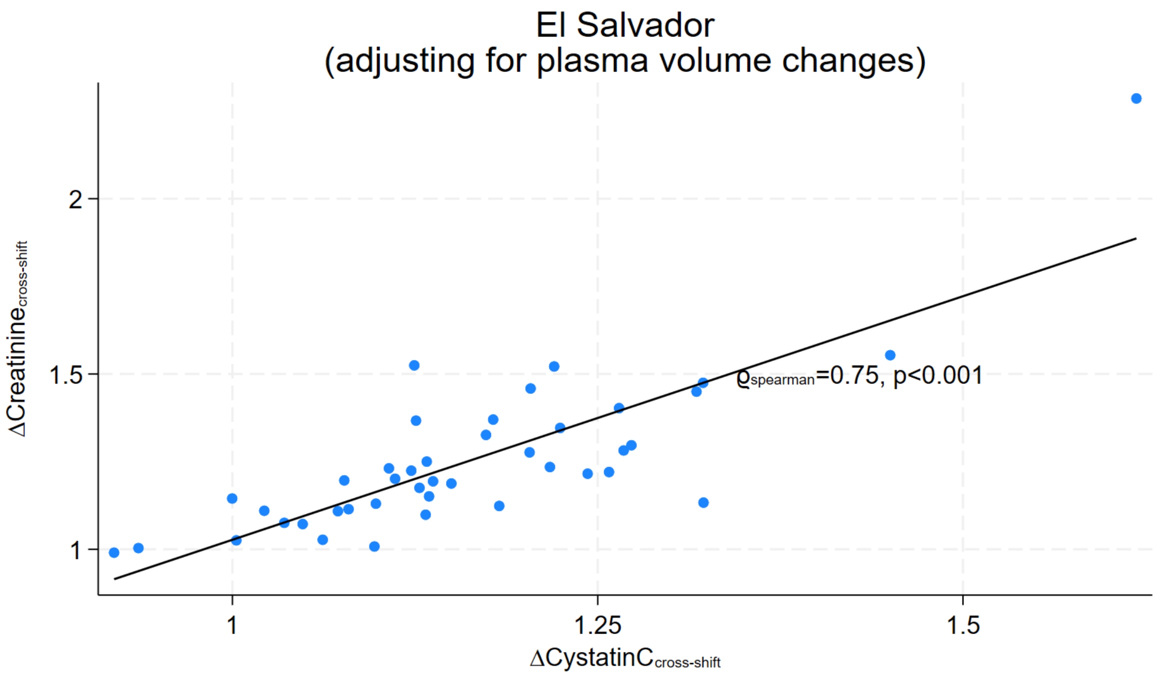

Supplement Figure 4. Associations between cross-shift changes in serum creatinine and cystatin C in the Salvadoran cohort, when adjusting for plasma volume changes using the Dill and Costill equation [8].

Supplement References

S1. Sorensen, C.J., J. Butler-Dawson, M. Dally et al., Risk Factors and Mechanisms Underlying Cross-shift Decline in Kidney Function in Guatemalan Sugarcane Workers. *J Occup Environ Med*. 2018;61(3):239-250. doi: 10.1097/JOM.0000000000001529.

S2. Butler-Dawson, J., L. Krisher, M. Dally et al., Sugarcane Workweek Study: Risk Factors for Daily Changes in Creatinine. *Kidney Int Rep*. 2021;6(9):2404–2414. doi: 10.1016/j.ekir.2021.06.003

S3. Andersson, A., E. Hansson, U. Ekström et al., Large difference but high correlation between creatinine and cystatin C estimated glomerular filtration rate in Mesoamerican sugarcane cutters. *Occup Environ Med*, 2022;79(7):497-502. doi: 10.1136/oemed-2021-107990.

S4. Lucas RAI, Skinner BD, Arias-Monge E et al. Targeting workload to ameliorate risk of heat stress in industrial sugarcane workers. *Scand J Work Environ Health*. 2023;49(1):43-52.

S5. Glaser J, Wegman DH, Arias-Monge E et al. Workplace Intervention for Heat Stress: Essential Elements of Design, Implementation, and Assessment. *Int J Environ Res Public Health.* 2022;19(7):3779.

S6. Theodorsson E, Berggren Söderlund M, eds., Laurells klinisk kemi i praktisk medicin, Lund, Sweden: Studentlitteratur; 2018.

S7. Alis R, Sanchis-Gomar F, Primo-Carrau C, et al. Hemoconcentration induced by exercise: Revisiting the Dill and Costill equation. *Scand J Med Sci Sports.* 2015;25(6):e630-e37. doi: https://doi.org/10.1111/sms.12393

S8. Griffin, B.R., J. Butler-Dawson, M. Dally et al., Unadjusted point of care creatinine results overestimate acute kidney injury incidence during field testing in Guatemala. *PLoS One.* 2018;13(9):e0204614.

S9. Hansson E, Jakobsson K, Glaser J, et al. Impact of heat and a rest-shade-hydration intervention program on productivity of piece-paid industrial agricultural workers at risk of chronic kidney disease of nontraditional origin. *Ann Work Expos Heal.* 2024:doi: 10.1093/annweh/wxae007

S10. Grubb A, Björk J, Nyman U, et al. Cystatin C, a marker for successful aging and glomerular filtration rate, is not influenced by inflammation. *Scand J Clin Lab Invest.* 2011;71(2):145-9. doi: 10.3109/00365513.2010.546879

S11. Singh D, Whooley MA, Ix JH, et al. Association of cystatin C and estimated GFR with inflammatory biomarkers: the Heart and Soul Study. *Nephrol Dial Transplant.* 2007;22(4):1087-92. doi: 10.1093/ndt/gfl744

S12. Hansson E, Wegman DH, Wesseling C, et al. Markers of kidney tubular and interstitial injury and function among sugarcane workers with cross-harvest serum creatinine elevation. *Occup Environ Med.* 2021:oemed-2021-107989. doi: 10.1136/oemed-2021-107989

S13. Hansson E, Wesseling C, Wegman D, et al. Point-of-care biomarkers for prediction of kidney function trajectory among sugarcane cutters: a comparative test accuracy study. *BMJ Open.* 2022;12(11):e060364. doi: 10.1136/bmjopen-2021-060364

S14. Caplin, B., K. Jakobsson, J. Glaser et al., International Collaboration for the Epidemiology of eGFR in Low and Middle Income Populations - Rationale and core protocol for the Disadvantaged Populations eGFR Epidemiology Study (DEGREE). *BMC Nephrol.* 2017. 18(1): p. 1.
